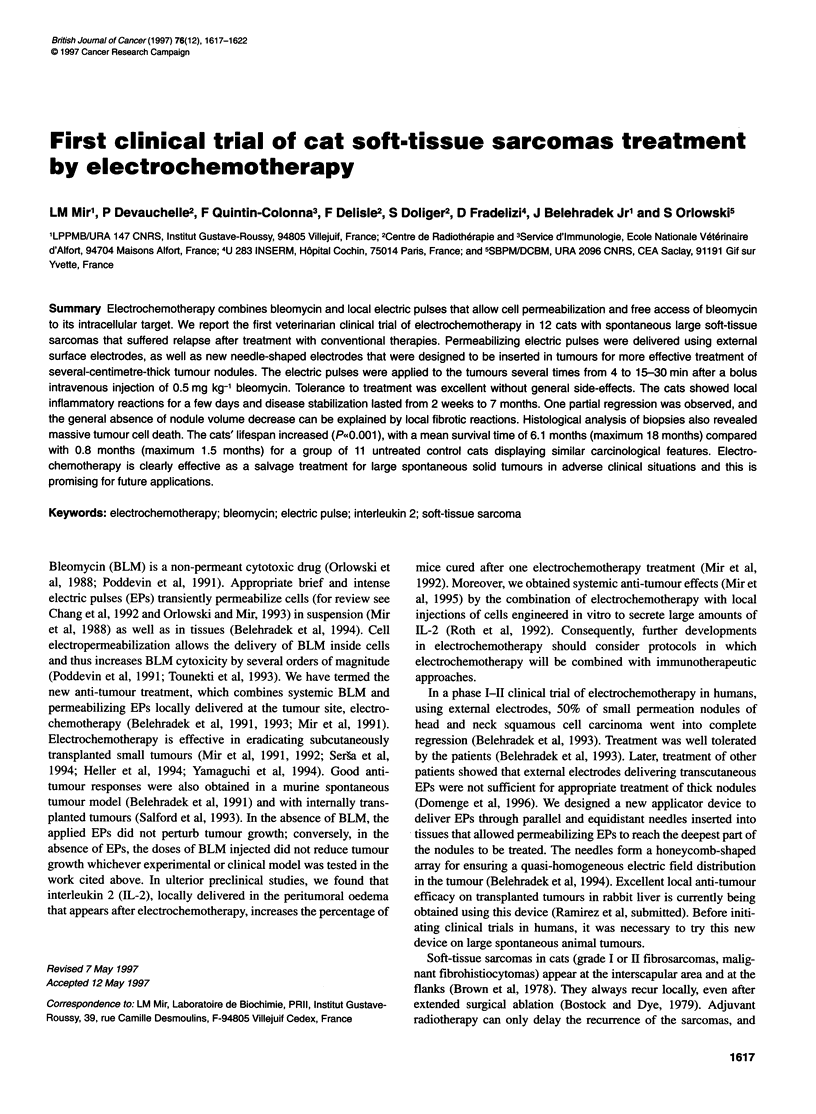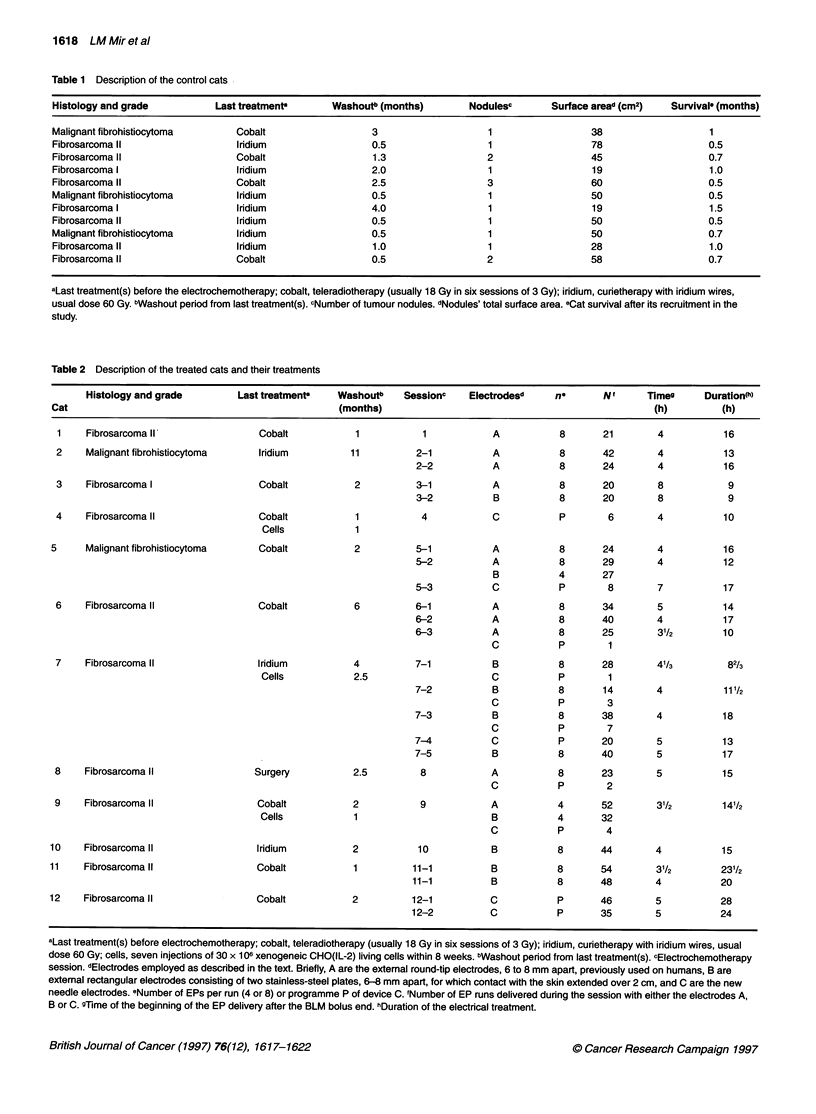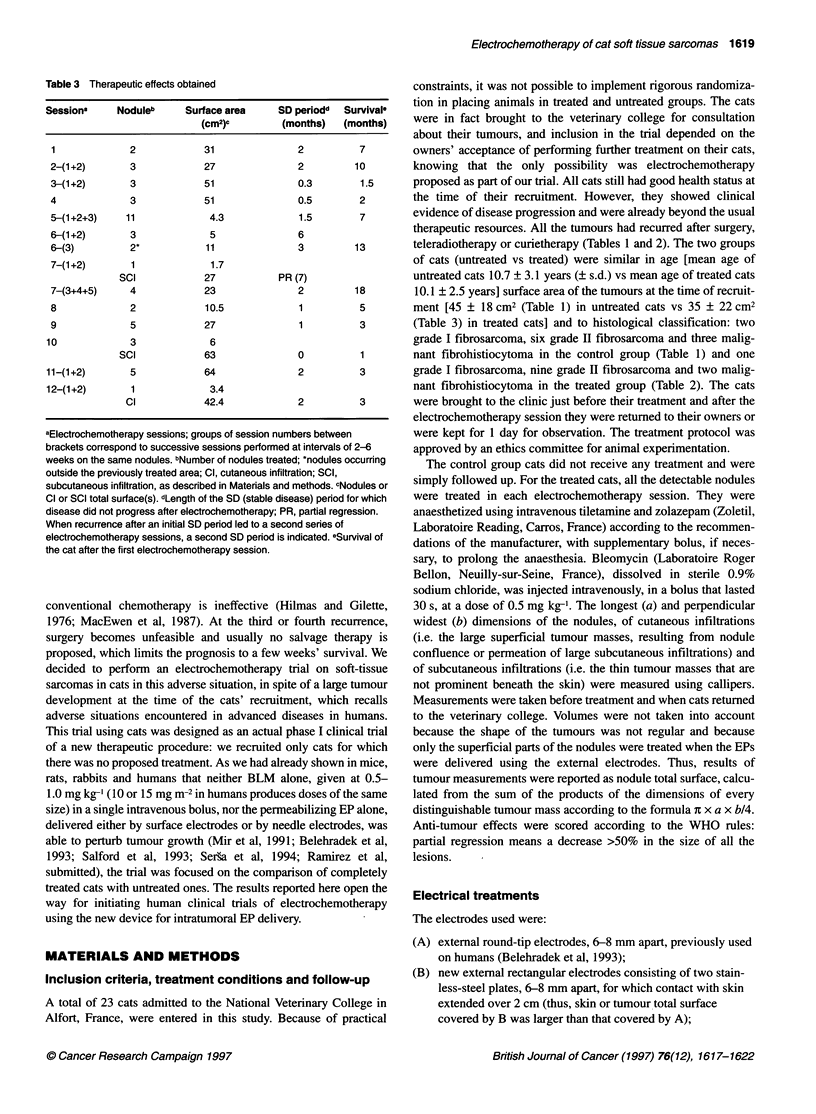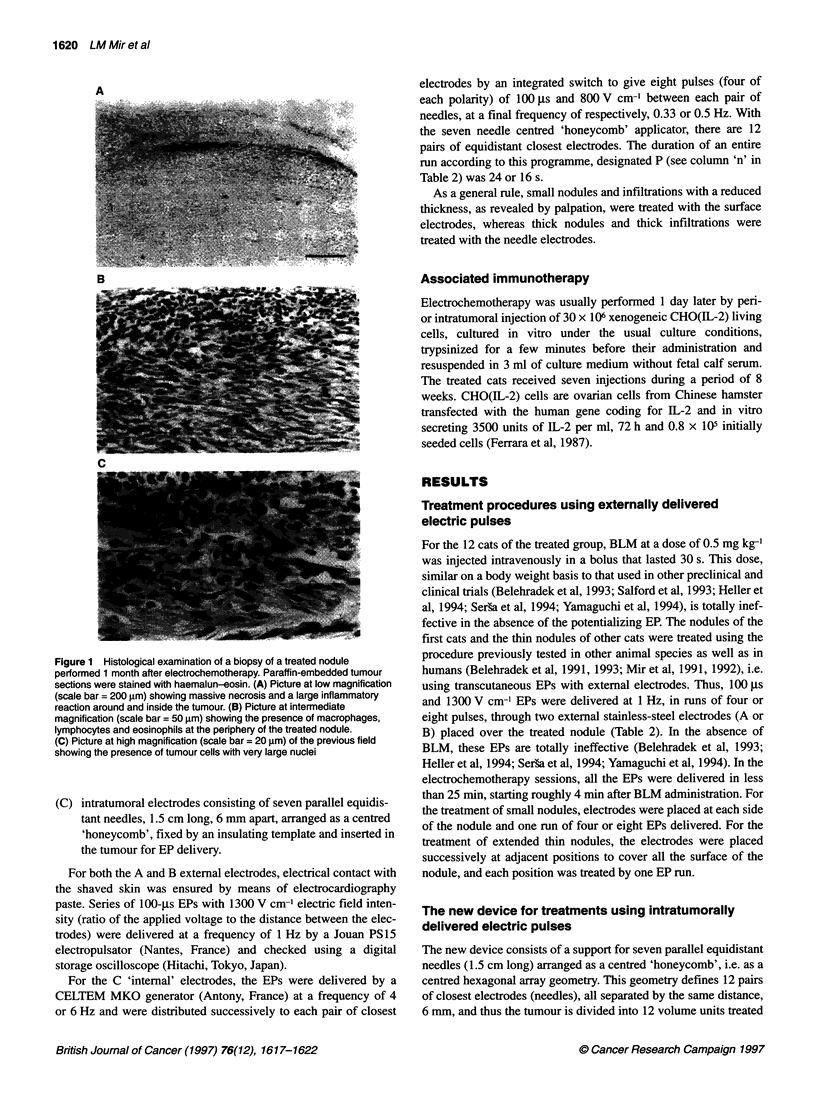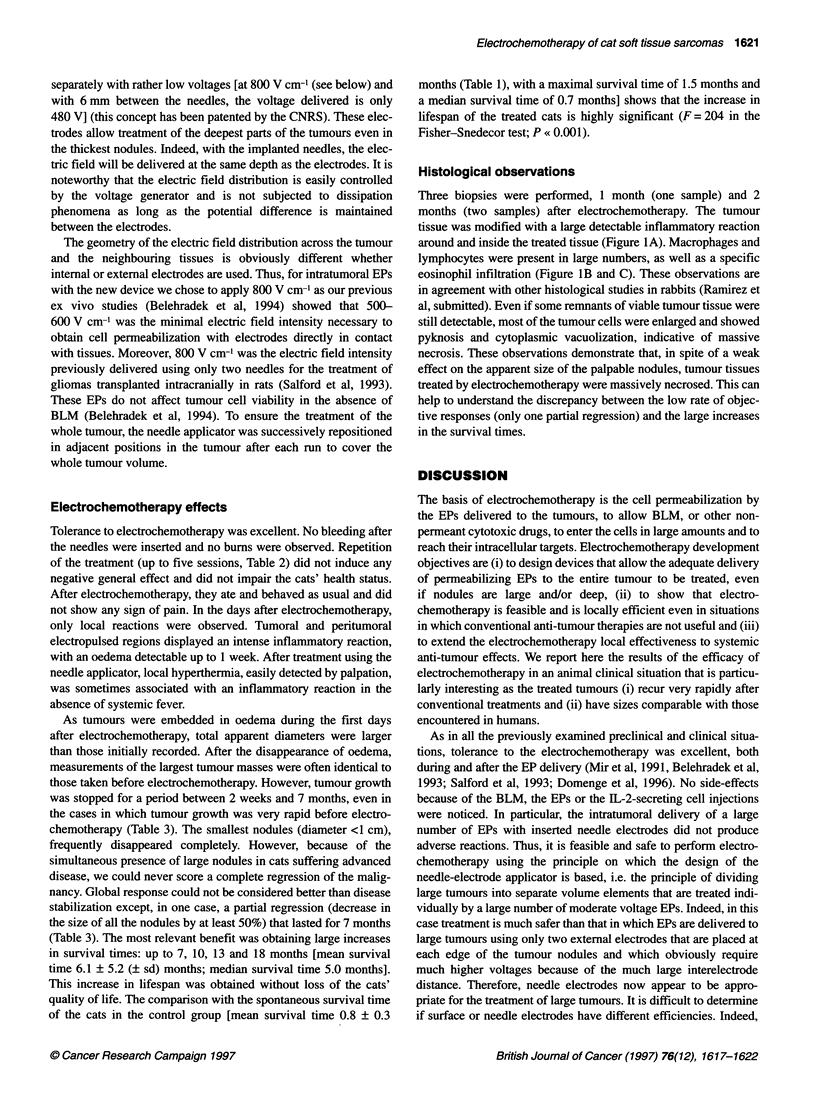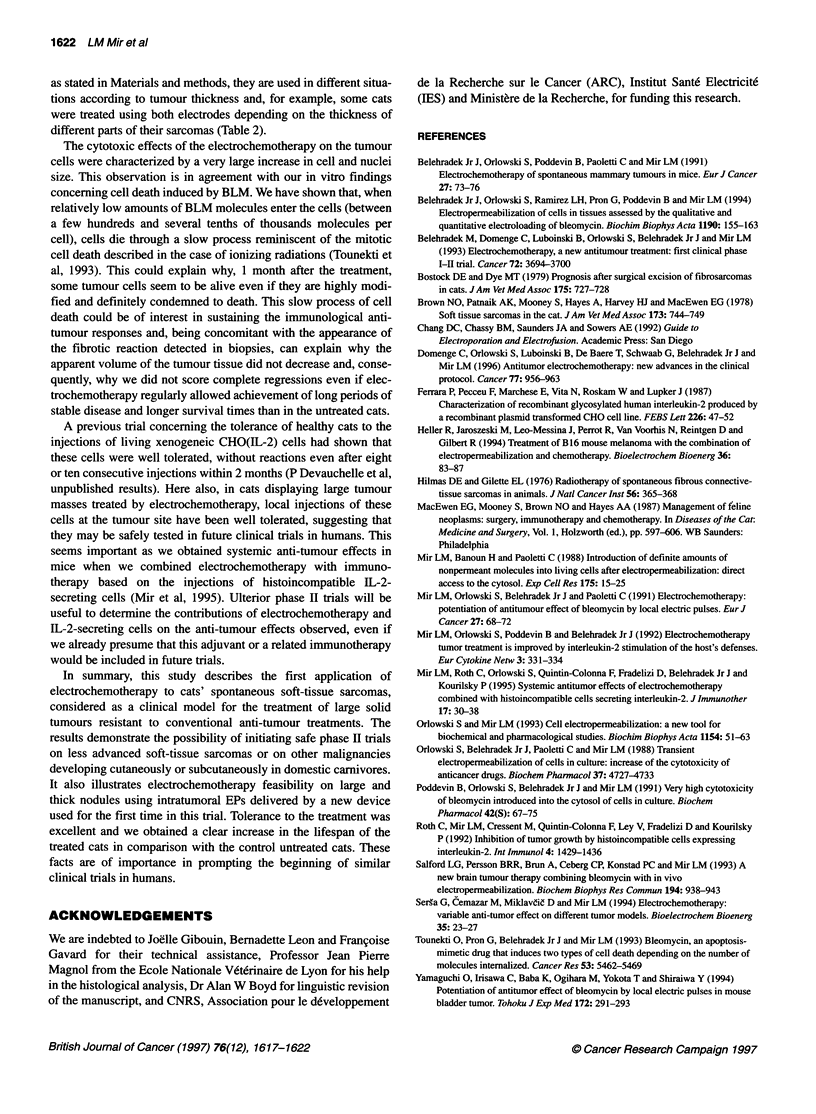# First clinical trial of cat soft-tissue sarcomas treatment by electrochemotherapy.

**DOI:** 10.1038/bjc.1997.606

**Published:** 1997

**Authors:** L. M. Mir, P. Devauchelle, F. Quintin-Colonna, F. Delisle, S. Doliger, D. Fradelizi, J. Belehradek, S. Orlowski

**Affiliations:** LPPMB/URA 147 CNRS, Institut Gustave-Roussy, Villejuif, France.

## Abstract

**Images:**


British Journal of Cancer (1997) 76(12), 1617–1622
© 1997 Cancer Research Campaign

### First clinical trial of cat soft-tissue sarcomas treatment by electrochemotherapy

**LM Mir[1], P Devauchelle[2], F Quintin-Colonna[3], F Delisle[2], S Doliger[2], D Fradelizi[4], J Belehradek Jr[1] and S Orlowski[5]**

[1]LPPMB/URA 147 CNRS, Institut Gustave-Roussy, 94805 Villejuif, France; [2]Centre de Radiothérapie and [3]Service d'Immunologie, Ecole Nationale Vétérinaire d'Alfort, 94704 Maisons Alfort, France; [4]U 283 INSERM, Hôpital Cochin, 75014 Paris, France; and [5]SBPM/DCBM, URA 2096 CNRS, CEA Saclay, 91191 Gif sur Yvette, France

**Summary** Electrochemotherapy combines bleomycin and local electric pulses that allow cell permeabilization and free access of bleomycin to its intracellular target. We report the first veterinarian clinical trial of electrochemotherapy in 12 cats with spontaneous large soft-tissue sarcomas that suffered relapse after treatment with conventional therapies. Permeabilizing electric pulses were delivered using external surface electrodes, as well as new needle-shaped electrodes that were designed to be inserted in tumours for more effective treatment of several-centimetre-thick tumour nodules. The electric pulses were applied to the tumours several times from 4 to 15–30 min after a bolus intravenous injection of 0.5 mg $kg^{-1}$ bleomycin. Tolerance to treatment was excellent without general side-effects. The cats showed local inflammatory reactions for a few days and disease stabilization lasted from 2 weeks to 7 months. One partial regression was observed, and the general absence of nodule volume decrease can be explained by local fibrotic reactions. Histological analysis of biopsies also revealed massive tumour cell death. The cats' lifespan increased ($P \ll 0.001$), with a mean survival time of 6.1 months (maximum 18 months) compared with 0.8 months (maximum 1.5 months) for a group of 11 untreated control cats displaying similar carcinological features. Electrochemotherapy is clearly effective as a salvage treatment for large spontaneous solid tumours in adverse clinical situations and this is promising for future applications.

**Keywords:** electrochemotherapy; bleomycin; electric pulse; interleukin 2; soft-tissue sarcoma

Bleomycin (BLM) is a non-permeant cytotoxic drug (Orlowski et al, 1988; Poddevin et al, 1991). Appropriate brief and intense electric pulses (EPs) transiently permeabilize cells (for review see Chang et al, 1992 and Orlowski and Mir, 1993) in suspension (Mir et al, 1988) as well as in tissues (Belehradek et al, 1994). Cell electropermeabilization allows the delivery of BLM inside cells and thus increases BLM cytoxicity by several orders of magnitude (Poddevin et al, 1991; Tounekti et al, 1993). We have termed the new anti-tumour treatment, which combines systemic BLM and permeabilizing EPs locally delivered at the tumour site, electrochemotherapy (Belehradek et al, 1991, 1993; Mir et al, 1991). Electrochemotherapy is effective in eradicating subcutaneously transplanted small tumours (Mir et al, 1991, 1992; Serša et al, 1994; Heller et al, 1994; Yamaguchi et al, 1994). Good anti-tumour responses were also obtained in a murine spontaneous tumour model (Belehradek et al, 1991) and with internally transplanted tumours (Salford et al, 1993). In the absence of BLM, the applied EPs did not perturb tumour growth; conversely, in the absence of EPs, the doses of BLM injected did not reduce tumour growth whichever experimental or clinical model was tested in the work cited above. In ulterior preclinical studies, we found that interleukin 2 (IL-2), locally delivered in the peritumoral oedema that appears after electrochemotherapy, increases the percentage of mice cured after one electrochemotherapy treatment (Mir et al, 1992). Moreover, we obtained systemic anti-tumour effects (Mir et al, 1995) by the combination of electrochemotherapy with local injections of cells engineered in vitro to secrete large amounts of IL-2 (Roth et al, 1992). Consequently, further developments in electrochemotherapy should consider protocols in which electrochemotherapy will be combined with immunotherapeutic approaches.

In a phase I–II clinical trial of electrochemotherapy in humans, using external electrodes, 50% of small permeation nodules of head and neck squamous cell carcinoma went into complete regression (Belehradek et al, 1993). Treatment was well tolerated by the patients (Belehradek et al, 1993). Later, treatment of other patients showed that external electrodes delivering transcutaneous EPs were not sufficient for appropriate treatment of thick nodules (Domenge et al, 1996). We designed a new applicator device to deliver EPs through parallel and equidistant needles inserted into tissues that allowed permeabilizing EPs to reach the deepest part of the nodules to be treated. The needles form a honeycomb-shaped array for ensuring a quasi-homogeneous electric field distribution in the tumour (Belehradek et al, 1994). Excellent local anti-tumour efficacy on transplanted tumours in rabbit liver is currently being obtained using this device (Ramirez et al, submitted). Before initiating clinical trials in humans, it was necessary to try this new device on large spontaneous animal tumours.

Soft-tissue sarcomas in cats (grade I or II fibrosarcomas, malignant fibrohistiocytomas) appear at the interscapular area and at the flanks (Brown et al, 1978). They always recur locally, even after extended surgical ablation (Bostock and Dye, 1979). Adjuvant radiotherapy can only delay the recurrence of the sarcomas, and

*Revised 7 May 1997*
*Accepted 12 May 1997*

*Correspondence to:* LM Mir, Laboratoire de Biochimie, PRII, Institut Gustave-Roussy, 39, rue Camille Desmoulins, F-94805 Villejuif Cedex, France

**Table 1** Description of the control cats

| Histology and grade | Last treatment[a] | Washout[b] (months) | Nodules[c] | Surface area[d] ($cm^2$) | Survival[e] (months) |
|---|---|---|---|---|---|
| Malignant fibrohistiocytoma | Cobalt | 3 | 1 | 38 | 1 |
| Fibrosarcoma II | Iridium | 0.5 | 1 | 78 | 0.5 |
| Fibrosarcoma II | Cobalt | 1.3 | 2 | 45 | 0.7 |
| Fibrosarcoma I | Iridium | 2.0 | 1 | 19 | 1.0 |
| Fibrosarcoma II | Cobalt | 2.5 | 3 | 60 | 0.5 |
| Malignant fibrohistiocytoma | Iridium | 0.5 | 1 | 50 | 0.5 |
| Fibrosarcoma I | Iridium | 4.0 | 1 | 19 | 1.5 |
| Fibrosarcoma II | Iridium | 0.5 | 1 | 50 | 0.5 |
| Malignant fibrohistiocytoma | Iridium | 0.5 | 1 | 50 | 0.7 |
| Fibrosarcoma II | Iridium | 1.0 | 1 | 28 | 1.0 |
| Fibrosarcoma II | Cobalt | 0.5 | 2 | 58 | 0.7 |

[a]Last treatment(s) before the electrochemotherapy; cobalt, teleradiotherapy (usually 18 Gy in six sessions of 3 Gy); iridium, curietherapy with iridium wires, usual dose 60 Gy. [b]Washout period from last treatment(s). [c]Number of tumour nodules. [d]Nodules' total surface area. [e]Cat survival after its recruitment in the study.

**Table 2** Description of the treated cats and their treatments

| Cat | Histology and grade | Last treatment[a] | Washout[b] (months) | Session[c] | Electrodes[d] | $n$[e] | $N$[f] | Time[g] (h) | Duration[(h)] (h) |
|---|---|---|---|---|---|---|---|---|---|
| 1 | Fibrosarcoma II | Cobalt | 1 | 1 | A | 8 | 21 | 4 | 16 |
| 2 | Malignant fibrohistiocytoma | Iridium | 11 | 2–1 | A | 8 | 42 | 4 | 13 |
| | | | | 2–2 | A | 8 | 24 | 4 | 16 |
| 3 | Fibrosarcoma I | Cobalt | 2 | 3–1 | A | 8 | 20 | 8 | 9 |
| | | | | 3–2 | B | 8 | 20 | 8 | 9 |
| 4 | Fibrosarcoma II | Cobalt | 1 | 4 | C | P | 6 | 4 | 10 |
| | | Cells | 1 | | | | | | |
| 5 | Malignant fibrohistiocytoma | Cobalt | 2 | 5–1 | A | 8 | 24 | 4 | 16 |
| | | | | 5–2 | A | 8 | 29 | 4 | 12 |
| | | | | | B | 4 | 27 | | |
| | | | | 5–3 | C | P | 8 | 7 | 17 |
| 6 | Fibrosarcoma II | Cobalt | 6 | 6–1 | A | 8 | 34 | 5 | 14 |
| | | | | 6–2 | A | 8 | 40 | 4 | 17 |
| | | | | 6–3 | A | 8 | 25 | 3½ | 10 |
| | | | | | C | P | 1 | | |
| 7 | Fibrosarcoma II | Iridium | 4 | 7–1 | B | 8 | 28 | 4⅓ | 8⅔ |
| | | Cells | 2.5 | | C | P | 1 | | |
| | | | | 7–2 | B | 8 | 14 | 4 | 11½ |
| | | | | | C | P | 3 | | |
| | | | | 7–3 | B | 8 | 38 | 4 | 18 |
| | | | | | C | P | 7 | | |
| | | | | 7–4 | C | P | 20 | 5 | 13 |
| | | | | 7–5 | B | 8 | 40 | 5 | 17 |
| 8 | Fibrosarcoma II | Surgery | 2.5 | 8 | A | 8 | 23 | 5 | 15 |
| | | | | | C | P | 2 | | |
| 9 | Fibrosarcoma II | Cobalt | 2 | 9 | A | 4 | 52 | 3½ | 14½ |
| | | Cells | 1 | | B | 4 | 32 | | |
| | | | | | C | P | 4 | | |
| 10 | Fibrosarcoma II | Iridium | 2 | 10 | B | 8 | 44 | 4 | 15 |
| 11 | Fibrosarcoma II | Cobalt | 1 | 11–1 | B | 8 | 54 | 3½ | 23½ |
| | | | | 11–1 | B | 8 | 48 | 4 | 20 |
| 12 | Fibrosarcoma II | Cobalt | 2 | 12–1 | C | P | 46 | 5 | 28 |
| | | | | 12–2 | C | P | 35 | 5 | 24 |

[a]Last treatment(s) before electrochemotherapy; cobalt, teleradiotherapy (usually 18 Gy in six sessions of 3 Gy); iridium, curietherapy with iridium wires, usual dose 60 Gy; cells, seven injections of 30 × $10^6$ xenogeneic CHO(IL-2) living cells within 8 weeks. [b]Washout period from last treatment(s). [c]Electrochemotherapy session. [d]Electrodes employed as described in the text. Briefly, A are the external round-tip electrodes, 6 to 8 mm apart, previously used on humans, B are external rectangular electrodes consisting of two stainless-steel plates, 6–8 mm apart, for which contact with the skin extended over 2 cm, and C are the new needle electrodes. [e]Number of EPs per run (4 or 8) or programme P of device C. [f]Number of EP runs delivered during the session with either the electrodes A, B or C. [g]Time of the beginning of the EP delivery after the BLM bolus end. [h]Duration of the electrical treatment.

 *© Cancer Research Campaign 1997*

**Table 3** Therapeutic effects obtained

| Session[a] | Nodule[b] | Surface area (cm²)[c] | SD period[d] (months) | Survival[e] (months) |
|---|---|---|---|---|
| 1 | 2 | 31 | 2 | 7 |
| 2–(1+2) | 3 | 27 | 2 | 10 |
| 3–(1+2) | 3 | 51 | 0.3 | 1.5 |
| 4 | 3 | 51 | 0.5 | 2 |
| 5–(1+2+3) | 11 | 4.3 | 1.5 | 7 |
| 6–(1+2) | 3 | 5 | 6 | |
| 6–(3) | 2* | 11 | 3 | 13 |
| 7–(1+2) | 1 | 1.7 | | |
| | SCI | 27 | PR (7) | |
| 7–(3+4+5) | 4 | 23 | 2 | 18 |
| 8 | 2 | 10.5 | 1 | 5 |
| 9 | 5 | 27 | 1 | 3 |
| 10 | 3 | 6 | | |
| | SCI | 63 | 0 | 1 |
| 11–(1+2) | 5 | 64 | 2 | 3 |
| 12–(1+2) | 1 | 3.4 | | |
| | CI | 42.4 | 2 | 3 |

[a]Electrochemotherapy sessions; groups of session numbers between brackets correspond to successive sessions performed at intervals of 2–6 weeks on the same nodules. [b]Number of nodules treated; *nodules occurring outside the previously treated area; CI, cutaneous infiltration; SCI, subcutaneous infiltration, as described in Materials and methods. [c]Nodules or CI or SCI total surface(s). [d]Length of the SD (stable disease) period for which disease did not progress after electrochemotherapy; PR, partial regression. When recurrence after an initial SD period led to a second series of electrochemotherapy sessions, a second SD period is indicated. [e]Survival of the cat after the first electrochemotherapy session.

conventional chemotherapy is ineffective (Hilmas and Gilette, 1976; MacEwen et al, 1987). At the third or fourth recurrence, surgery becomes unfeasible and usually no salvage therapy is proposed, which limits the prognosis to a few weeks' survival. We decided to perform an electrochemotherapy trial on soft-tissue sarcomas in cats in this adverse situation, in spite of a large tumour development at the time of the cats' recruitment, which recalls adverse situations encountered in advanced diseases in humans. This trial using cats was designed as an actual phase I clinical trial of a new therapeutic procedure: we recruited only cats for which there was no proposed treatment. As we had already shown in mice, rats, rabbits and humans that neither BLM alone, given at 0.5–1.0 mg $kg^{-1}$ (10 or 15 mg $m^{-2}$ in humans produces doses of the same size) in a single intravenous bolus, nor the permeabilizing EP alone, delivered either by surface electrodes or by needle electrodes, was able to perturb tumour growth (Mir et al, 1991; Belehradek et al, 1993; Salford et al, 1993; Serša et al, 1994; Ramirez et al, submitted), the trial was focused on the comparison of completely treated cats with untreated ones. The results reported here open the way for initiating human clinical trials of electrochemotherapy using the new device for intratumoral EP delivery.

### MATERIALS AND METHODS

### Inclusion criteria, treatment conditions and follow-up

A total of 23 cats admitted to the National Veterinary College in Alfort, France, were entered in this study. Because of practical constraints, it was not possible to implement rigorous randomization in placing animals in treated and untreated groups. The cats were in fact brought to the veterinary college for consultation about their tumours, and inclusion in the trial depended on the owners' acceptance of performing further treatment on their cats, knowing that the only possibility was electrochemotherapy proposed as part of our trial. All cats still had good health status at the time of their recruitment. However, they showed clinical evidence of disease progression and were already beyond the usual therapeutic resources. All the tumours had recurred after surgery, teleradiotherapy or curietherapy (Tables 1 and 2). The two groups of cats (untreated vs treated) were similar in age [mean age of untreated cats 10.7 ± 3.1 years (± s.d.) vs mean age of treated cats 10.1 ± 2.5 years] surface area of the tumours at the time of recruitment [45 ± 18 $cm^2$ (Table 1) in untreated cats vs 35 ± 22 $cm^2$ (Table 3) in treated cats] and to histological classification: two grade I fibrosarcoma, six grade II fibrosarcoma and three malignant fibrohistiocytoma in the control group (Table 1) and one grade I fibrosarcoma, nine grade II fibrosarcoma and two malignant fibrohistiocytoma in the treated group (Table 2). The cats were brought to the clinic just before their treatment and after the electrochemotherapy session they were returned to their owners or were kept for 1 day for observation. The treatment protocol was approved by an ethics committee for animal experimentation.

The control group cats did not receive any treatment and were simply followed up. For the treated cats, all the detectable nodules were treated in each electrochemotherapy session. They were anaesthetized using intravenous tiletamine and zolazepam (Zoletil, Laboratoire Reading, Carros, France) according to the recommendations of the manufacturer, with supplementary bolus, if necessary, to prolong the anaesthesia. Bleomycin (Laboratoire Roger Bellon, Neuilly-sur-Seine, France), dissolved in sterile 0.9% sodium chloride, was injected intravenously, in a bolus that lasted 30 s, at a dose of 0.5 mg $kg^{-1}$. The longest ($a$) and perpendicular widest ($b$) dimensions of the nodules, of cutaneous infiltrations (i.e. the large superficial tumour masses, resulting from nodule confluence or permeation of large subcutaneous infiltrations) and of subcutaneous infiltrations (i.e. the thin tumour masses that are not prominent beneath the skin) were measured using callipers. Measurements were taken before treatment and when cats returned to the veterinary college. Volumes were not taken into account because the shape of the tumours was not regular and because only the superficial parts of the nodules were treated when the EPs were delivered using the external electrodes. Thus, results of tumour measurements were reported as nodule total surface, calculated from the sum of the products of the dimensions of every distinguishable tumour mass according to the formula $\pi \times a \times b/4$. Anti-tumour effects were scored according to the WHO rules: partial regression means a decrease >50% in the size of all the lesions.

### Electrical treatments

The electrodes used were:

(A) external round-tip electrodes, 6–8 mm apart, previously used on humans (Belehradek et al, 1993);
(B) new external rectangular electrodes consisting of two stainless-steel plates, 6–8 mm apart, for which contact with skin extended over 2 cm (thus, skin or tumour total surface covered by B was larger than that covered by A);

© Cancer Research Campaign 1997

**Figure 1** Histological examination of a biopsy of a treated nodule performed 1 month after electrochemotherapy. Paraffin-embedded tumour sections were stained with haemalun–eosin. (**A**) Picture at low magnification (scale bar = 200 μm) showing massive necrosis and a large inflammatory reaction around and inside the tumour. (**B**) Picture at intermediate magnification (scale bar = 50 μm) showing the presence of macrophages, lymphocytes and eosinophils at the periphery of the treated nodule. (**C**) Picture at high magnification (scale bar = 20 μm) of the previous field showing the presence of tumour cells with very large nuclei

(C) intratumoral electrodes consisting of seven parallel equidistant needles, 1.5 cm long, 6 mm apart, arranged as a centred 'honeycomb', fixed by an insulating template and inserted in the tumour for EP delivery.

For both the A and B external electrodes, electrical contact with the shaved skin was ensured by means of electrocardiography paste. Series of 100-μs EPs with 1300 V $cm^{-1}$ electric field intensity (ratio of the applied voltage to the distance between the electrodes) were delivered at a frequency of 1 Hz by a Jouan PS15 electropulsator (Nantes, France) and checked using a digital storage oscilloscope (Hitachi, Tokyo, Japan).

For the C 'internal' electrodes, the EPs were delivered by a CELTEM MKO generator (Antony, France) at a frequency of 4 or 6 Hz and were distributed successively to each pair of closest electrodes by an integrated switch to give eight pulses (four of each polarity) of 100 μs and 800 V $cm^{-1}$ between each pair of needles, at a final frequency of respectively, 0.33 or 0.5 Hz. With the seven needle centred 'honeycomb' applicator, there are 12 pairs of equidistant closest electrodes. The duration of an entire run according to this programme, designated P (see column 'n' in Table 2) was 24 or 16 s.

As a general rule, small nodules and infiltrations with a reduced thickness, as revealed by palpation, were treated with the surface electrodes, whereas thick nodules and thick infiltrations were treated with the needle electrodes.

### Associated immunotherapy

Electrochemotherapy was usually performed 1 day later by peri- or intratumoral injection of $30 \times 10^6$ xenogeneic CHO(IL-2) living cells, cultured in vitro under the usual culture conditions, trypsinized for a few minutes before their administration and resuspended in 3 ml of culture medium without fetal calf serum. The treated cats received seven injections during a period of 8 weeks. CHO(IL-2) cells are ovarian cells from Chinese hamster transfected with the human gene coding for IL-2 and in vitro secreting 3500 units of IL-2 per ml, 72 h and $0.8 \times 10^5$ initially seeded cells (Ferrara et al, 1987).

### RESULTS

### Treatment procedures using externally delivered electric pulses

For the 12 cats of the treated group, BLM at a dose of 0.5 mg $kg^{-1}$ was injected intravenously in a bolus that lasted 30 s. This dose, similar on a body weight basis to that used in other preclinical and clinical trials (Belehradek et al, 1993; Salford et al, 1993; Heller et al, 1994; Serša et al, 1994; Yamaguchi et al, 1994), is totally ineffective in the absence of the potentializing EP. The nodules of the first cats and the thin nodules of other cats were treated using the procedure previously tested in other animal species as well as in humans (Belehradek et al, 1991, 1993; Mir et al, 1991, 1992), i.e. using transcutaneous EPs with external electrodes. Thus, 100 μs and 1300 V $cm^{-1}$ EPs were delivered at 1 Hz, in runs of four or eight pulses, through two external stainless-steel electrodes (A or B) placed over the treated nodule (Table 2). In the absence of BLM, these EPs are totally ineffective (Belehradek et al, 1993; Heller et al, 1994; Serša et al, 1994; Yamaguchi et al, 1994). In the electrochemotherapy sessions, all the EPs were delivered in less than 25 min, starting roughly 4 min after BLM administration. For the treatment of small nodules, electrodes were placed at each side of the nodule and one run of four or eight EPs delivered. For the treatment of extended thin nodules, the electrodes were placed successively at adjacent positions to cover all the surface of the nodule, and each position was treated by one EP run.

### The new device for treatments using intratumorally delivered electric pulses

The new device consists of a support for seven parallel equidistant needles (1.5 cm long) arranged as a centred 'honeycomb', i.e. as a centred hexagonal array geometry. This geometry defines 12 pairs of closest electrodes (needles), all separated by the same distance, 6 mm, and thus the tumour is divided into 12 volume units treated

© Cancer Research Campaign 1997

separately with rather low voltages [at 800 V $cm^{-1}$ (see below) and with 6 mm between the needles, the voltage delivered is only 480 V] (this concept has been patented by the CNRS). These electrodes allow treatment of the deepest parts of the tumours even in the thickest nodules. Indeed, with the implanted needles, the electric field will be delivered at the same depth as the electrodes. It is noteworthy that the electric field distribution is easily controlled by the voltage generator and is not subjected to dissipation phenomena as long as the potential difference is maintained between the electrodes.

The geometry of the electric field distribution across the tumour and the neighbouring tissues is obviously different whether internal or external electrodes are used. Thus, for intratumoral EPs with the new device we chose to apply 800 V $cm^{-1}$ as our previous ex vivo studies (Belehradek et al, 1994) showed that 500–600 V $cm^{-1}$ was the minimal electric field intensity necessary to obtain cell permeabilization with electrodes directly in contact with tissues. Moreover, 800 V $cm^{-1}$ was the electric field intensity previously delivered using only two needles for the treatment of gliomas transplanted intracranially in rats (Salford et al, 1993). These EPs do not affect tumour cell viability in the absence of BLM (Belehradek et al, 1994). To ensure the treatment of the whole tumour, the needle applicator was successively repositioned in adjacent positions in the tumour after each run to cover the whole tumour volume.

### Electrochemotherapy effects

Tolerance to electrochemotherapy was excellent. No bleeding after the needles were inserted and no burns were observed. Repetition of the treatment (up to five sessions, Table 2) did not induce any negative general effect and did not impair the cats' health status. After electrochemotherapy, they ate and behaved as usual and did not show any sign of pain. In the days after electrochemotherapy, only local reactions were observed. Tumoral and peritumoral electropulsed regions displayed an intense inflammatory reaction, with an oedema detectable up to 1 week. After treatment using the needle applicator, local hyperthermia, easily detected by palpation, was sometimes associated with an inflammatory reaction in the absence of systemic fever.

As tumours were embedded in oedema during the first days after electrochemotherapy, total apparent diameters were larger than those initially recorded. After the disappearance of oedema, measurements of the largest tumour masses were often identical to those taken before electrochemotherapy. However, tumour growth was stopped for a period between 2 weeks and 7 months, even in the cases in which tumour growth was very rapid before electrochemotherapy (Table 3). The smallest nodules (diameter <1 cm), frequently disappeared completely. However, because of the simultaneous presence of large nodules in cats suffering advanced disease, we could never score a complete regression of the malignancy. Global response could not be considered better than disease stabilization except, in one case, a partial regression (decrease in the size of all the nodules by at least 50%) that lasted for 7 months (Table 3). The most relevant benefit was obtaining large increases in survival times: up to 7, 10, 13 and 18 months [mean survival time 6.1 ± 5.2 (± sd) months; median survival time 5.0 months]. This increase in lifespan was obtained without loss of the cats' quality of life. The comparison with the spontaneous survival time of the cats in the control group [mean survival time 0.8 ± 0.3 months (Table 1), with a maximal survival time of 1.5 months and a median survival time of 0.7 months] shows that the increase in lifespan of the treated cats is highly significant ($F = 204$ in the Fisher–Snedecor test; $P \ll 0.001$).

### Histological observations

Three biopsies were performed, 1 month (one sample) and 2 months (two samples) after electrochemotherapy. The tumour tissue was modified with a large detectable inflammatory reaction around and inside the treated tissue (Figure 1A). Macrophages and lymphocytes were present in large numbers, as well as a specific eosinophil infiltration (Figure 1B and C). These observations are in agreement with other histological studies in rabbits (Ramirez et al, submitted). Even if some remnants of viable tumour tissue were still detectable, most of the tumour cells were enlarged and showed pyknosis and cytoplasmic vacuolization, indicative of massive necrosis. These observations demonstrate that, in spite of a weak effect on the apparent size of the palpable nodules, tumour tissues treated by electrochemotherapy were massively necrosed. This can help to understand the discrepancy between the low rate of objective responses (only one partial regression) and the large increases in the survival times.

### DISCUSSION

The basis of electrochemotherapy is the cell permeabilization by the EPs delivered to the tumours, to allow BLM, or other non-permeant cytotoxic drugs, to enter the cells in large amounts and to reach their intracellular targets. Electrochemotherapy development objectives are (i) to design devices that allow the adequate delivery of permeabilizing EPs to the entire tumour to be treated, even if nodules are large and/or deep, (ii) to show that electrochemotherapy is feasible and is locally efficient even in situations in which conventional anti-tumour therapies are not useful and (iii) to extend the electrochemotherapy local effectiveness to systemic anti-tumour effects. We report here the results of the efficacy of electrochemotherapy in an animal clinical situation that is particularly interesting as the treated tumours (i) recur very rapidly after conventional treatments and (ii) have sizes comparable with those encountered in humans.

As in all the previously examined preclinical and clinical situations, tolerance to the electrochemotherapy was excellent, both during and after the EP delivery (Mir et al, 1991, Belehradek et al, 1993; Salford et al, 1993; Domenge et al, 1996). No side-effects because of the BLM, the EPs or the IL-2-secreting cell injections were noticed. In particular, the intratumoral delivery of a large number of EPs with inserted needle electrodes did not produce adverse reactions. Thus, it is feasible and safe to perform electrochemotherapy using the principle on which the design of the needle-electrode applicator is based, i.e. the principle of dividing large tumours into separate volume elements that are treated individually by a large number of moderate voltage EPs. Indeed, in this case treatment is much safer than that in which EPs are delivered to large tumours using only two external electrodes that are placed at each edge of the tumour nodules and which obviously require much higher voltages because of the much large interelectrode distance. Therefore, needle electrodes now appear to be appropriate for the treatment of large tumours. It is difficult to determine if surface or needle electrodes have different efficiencies. Indeed,

© Cancer Research Campaign 1997

as stated in Materials and methods, they are used in different situations according to tumour thickness and, for example, some cats were treated using both electrodes depending on the thickness of different parts of their sarcomas (Table 2).

The cytotoxic effects of the electrochemotherapy on the tumour cells were characterized by a very large increase in cell and nuclei size. This observation is in agreement with our in vitro findings concerning cell death induced by BLM. We have shown that, when relatively low amounts of BLM molecules enter the cells (between a few hundreds and several tenths of thousands molecules per cell), cells die through a slow process reminiscent of the mitotic cell death described in the case of ionizing radiations (Tounekti et al, 1993). This could explain why, 1 month after the treatment, some tumour cells seem to be alive even if they are highly modified and definitely condemned to death. This slow process of cell death could be of interest in sustaining the immunological anti-tumour responses and, being concomitant with the appearance of the fibrotic reaction detected in biopsies, can explain why the apparent volume of the tumour tissue did not decrease and, consequently, why we did not score complete regressions even if electrochemotherapy regularly allowed achievement of long periods of stable disease and longer survival times than in the untreated cats.

A previous trial concerning the tolerance of healthy cats to the injections of living xenogeneic CHO(IL-2) cells had shown that these cells were well tolerated, without reactions even after eight or ten consecutive injections within 2 months (P Devauchelle et al, unpublished results). Here also, in cats displaying large tumour masses treated by electrochemotherapy, local injections of these cells at the tumour site have been well tolerated, suggesting that they may be safely tested in future clinical trials in humans. This seems important as we obtained systemic anti-tumour effects in mice when we combined electrochemotherapy with immunotherapy based on the injections of histoincompatible IL-2-secreting cells (Mir et al, 1995). Ulterior phase II trials will be useful to determine the contributions of electrochemotherapy and IL-2-secreting cells on the anti-tumour effects observed, even if we already presume that this adjuvant or a related immunotherapy would be included in future trials.

In summary, this study describes the first application of electrochemotherapy to cats' spontaneous soft-tissue sarcomas, considered as a clinical model for the treatment of large solid tumours resistant to conventional anti-tumour treatments. The results demonstrate the possibility of initiating safe phase II trials on less advanced soft-tissue sarcomas or on other malignancies developing cutaneously or subcutaneously in domestic carnivores. It also illustrates electrochemotherapy feasibility on large and thick nodules using intratumoral EPs delivered by a new device used for the first time in this trial. Tolerance to the treatment was excellent and we obtained a clear increase in the lifespan of the treated cats in comparison with the control untreated cats. These facts are of importance in prompting the beginning of similar clinical trials in humans.

### ACKNOWLEDGEMENTS

We are indebted to Joëlle Gibouin, Bernadette Leon and Françoise Gavard for their technical assistance, Professor Jean Pierre Magnol from the Ecole Nationale Vétérinaire de Lyon for his help in the histological analysis, Dr Alan W Boyd for linguistic revision of the manuscript, and CNRS, Association pour le développement de la Recherche sur le Cancer (ARC), Institut Santé Electricité (IES) and Ministère de la Recherche, for funding this research.

### REFERENCES

Belehradek Jr J, Orlowski S, Poddevin B, Paoletti C and Mir LM (1991) Electrochemotherapy of spontaneous mammary tumours in mice. *Eur J Cancer* **27**: 73–76

Belehradek Jr J, Orlowski S, Ramirez LH, Pron G, Poddevin B and Mir LM (1994) Electropermeabilization of cells in tissues assessed by the qualitative and quantitative electroloading of bleomycin. *Biochim Biophys Acta* **1190**: 155–163

Belehradek M, Domenge C, Luboinski B, Orlowski S, Belehradek Jr J and Mir LM (1993) Electrochemotherapy, a new antitumour treatment: first clinical phase I–II trial. *Cancer* **72**: 3694–3700

Bostock DE and Dye MT (1979) Prognosis after surgical excision of fibrosarcomas in cats. *J Am Vet Med Assoc* **175**: 727–728

Brown NO, Patnaik AK, Mooney S, Hayes A, Harvey HJ and MacEwen EG (1978) Soft tissue sarcomas in the cat. *J Am Vet Med Assoc* **173**: 744–749

Chang DC, Chassy BM, Saunders JA and Sowers AE (1992) *Guide to Electroporation and Electrofusion*. Academic Press: San Diego

Domenge C, Orlowski S, Luboinski B, De Baere T, Schwaab G, Belehradek Jr J and Mir LM (1996) Antitumor electrochemotherapy: new advances in the clinical protocol. *Cancer* **77**: 956–963

Ferrara P, Pecceu F, Marchese E, Vita N, Roskam W and Lupker J (1987) Characterization of recombinant glycosylated human interleukin-2 produced by a recombinant plasmid transformed CHO cell line. *FEBS Lett* **226**: 47–52

Heller R, Jaroszeski M, Leo-Messina J, Perrot R, Van Voorhis N, Reintgen D and Gilbert R (1994) Treatment of B16 mouse melanoma with the combination of electropermeabilization and chemotherapy. *Bioelectrochem Bioenerg* **36**: 83–87

Hilmas DE and Gilette EL (1976) Radiotherapy of spontaneous fibrous connective-tissue sarcomas in animals. *J Natl Cancer Inst* **56**: 365–368

MacEwen EG, Mooney S, Brown NO and Hayes AA (1987) Management of feline neoplasms: surgery, immunotherapy and chemotherapy. In *Diseases of the Cat: Medicine and Surgery*, Vol. 1, Holzworth (ed.), pp. 597–606. WB Saunders: Philadelphia

Mir LM, Banoun H and Paoletti C (1988) Introduction of definite amounts of nonpermeant molecules into living cells after electropermeabilization: direct access to the cytosol. *Exp Cell Res* **175**: 15–25

Mir LM, Orlowski S, Belehradek Jr J and Paoletti C (1991) Electrochemotherapy: potentiation of antitumour effect of bleomycin by local electric pulses. *Eur J Cancer* **27**: 68–72

Mir LM, Orlowski S, Poddevin B and Belehradek Jr J (1992) Electrochemotherapy tumor treatment is improved by interleukin-2 stimulation of the host's defenses. *Eur Cytokine Netw* **3**: 331–334

Mir LM, Roth C, Orlowski S, Quintin-Colonna F, Fradelizi D, Belehradek Jr J and Kourilsky P (1995) Systemic antitumor effects of electrochemotherapy combined with histoincompatible cells secreting interleukin-2. *J Immunother* **17**: 30–38

Orlowski S and Mir LM (1993) Cell electropermeabilization: a new tool for biochemical and pharmacological studies. *Biochim Biophys Acta* **1154**: 51–63

Orlowski S, Belehradek Jr J, Paoletti C and Mir LM (1988) Transient electropermeabilization of cells in culture: increase of the cytotoxicity of anticancer drugs. *Biochem Pharmacol* **37**: 4727–4733

Poddevin B, Orlowski S, Belehradek Jr J and Mir LM (1991) Very high cytotoxicity of bleomycin introduced into the cytosol of cells in culture. *Biochem Pharmacol* **42(S)**: 67–75

Roth C, Mir LM, Cressent M, Quintin-Colonna F, Ley V, Fradelizi D and Kourilsky P (1992) Inhibition of tumor growth by histoincompatible cells expressing interleukin-2. *Int Immunol* **4**: 1429–1436

Salford LG, Persson BRR, Brun A, Ceberg CP, Konstad PC and Mir LM (1993) A new brain tumour therapy combining bleomycin with in vivo electropermeabilization. *Biochem Biophys Res Commun* **194**: 938–943

Serša G, Čemazar M, Miklavčič D and Mir LM (1994) Electrochemotherapy: variable anti-tumor effect on different tumor models. *Bioelectrochem Bioenerg* **35**: 23–27

Tounekti O, Pron G, Belehradek Jr J and Mir LM (1993) Bleomycin, an apoptosis-mimetic drug that induces two types of cell death depending on the number of molecules internalized. *Cancer Res* **53**: 5462–5469

Yamaguchi O, Irisawa C, Baba K, Ogihara M, Yokota T and Shiraiwa Y (1994) Potentiation of antitumor effect of bleomycin by local electric pulses in mouse bladder tumor. *Tohoku J Exp Med* **172**: 291–293

© Cancer Research Campaign 1997